# Early goal-directed therapy in the management of severe sepsis or septic shock in adults: a meta-analysis of randomized controlled trials

**DOI:** 10.1186/s12916-015-0312-9

**Published:** 2015-04-03

**Authors:** Ling Zhang, Guijun Zhu, Li Han, Ping Fu

**Affiliations:** Division of Nephrology and Intensive Care Medicine, West China Hospital of Sichuan University, Chengdu, Sichuan China; Division of Intensive Care Unit, Fourth Hospital of Hebei Medical University, Shijiazhuang, Hebei China; Division of Intensive Care Unit, West China Hospital of Sichuan University, Chengdu, Sichuan China; Division of Nephrology, West China Hospital of Sichuan University, Chengdu, Sichuan China

**Keywords:** EGDT, Early goal-directed, Resuscitation, Sepsis, Meta-analysis

## Abstract

**Background:**

The Surviving Sepsis Campaign guidelines have proposed early goal-directed therapy (EGDT) as a key strategy to decrease mortality among patients with severe sepsis or septic shock. However, its effectiveness is uncertain.

**Methods:**

We searched for relevant studies in Medline, Embase, the Cochrane Library, Google Scholar, and a Chinese database (SinoMed), as well as relevant references from January 1966 to October 2014. We performed a systematic review and meta-analysis of all eligible randomized controlled trials (RCTs) of EGDT for patients with severe sepsis or septic shock. The primary outcome was mortality; secondary outcomes were length of ICU and in-hospital stay, mechanical ventilation support, vasopressor and inotropic agents support, fluid administration, and red cell transfusion. We pooled relative risks (RRs) or weighted mean differences (MDs) with 95% confidence intervals (95% CI) using Review Manager 5.2.

**Results:**

We included 10 RCTs from 2001 to 2014 involving 4,157 patients. Pooled analyses of all studies showed no significant difference in mortality between the EGDT and the control group (RR 0.91, 95% CI: 0.79 to 1.04, *P* = 0.17), with substantial heterogeneity (*χ*2 = 23.65, *I*^2^ = 58%). In the subgroup analysis, standard EGDT, but not modified EGDT, was associated with lower mortality rate in comparison with the usual care group (RR 0.84, 95%CI: 0.72 to 0.98, *P* = 0.03). However, EGDT was associated with a higher mortality rate in comparison with the early lactate clearance group (RR 1.52, 95% CI: 1.06 to 2.18, *P* = 0.02). In the first 6 h, compared with usual care, patients in EGDT received more inotropic agents (*P* = 0.04), fluid administration (*P* = 0.05), and red cell transfusion (*P* < 0.01). There were no significant differences in length of ICU stay (*P* = 0.73) or in-hospital stay (*P* = 0.57), ventilation rate (*P* = 0.53), and vasopressor support (*P* = 0.63).

**Conclusions:**

EGDT was not associated with a survival benefit among patients with severe sepsis or septic shock. Instead, EGDT was associated with a higher mortality rate in comparison to the early lactate clearance group. Further high-quality RCTs comparing EGDT with early lactate clearance are desirable.

## Background

Severe sepsis and septic shock are common complications of patients with critical illness, with an annual incidence of up to 300 cases per 100,000 people in the United States [1]. Despite efforts to improve its management, sepsis remains the 10th leading cause of death in the United States, with an associated mortality of 20% to 50% [1-3]. In 2001, Rivers et al. first reported that a specific 6-h protocol of early goal-directed therapy (EGDT) significantly reduced the mortality rate of patients with severe sepsis and septic shock presenting to the emergency department, as compared with the usual therapy [4]. EGDT was subsequently incorporated into the 6-h resuscitation bundle of the Surviving Sepsis Campaign guidelines [5-7], and many studies showed a survival benefit with EGDT or a sepsis bundle including EGDT [8-12]. However, in 2014, two multicenter randomized controlled trials (RCTs) showed that EGDT was not associated with a survival benefit in comparison with usual care [13,14]. A recent cohort study showed that EGDT might increase the risk of fluid overload and mortality [15]. Thus, we sought to systematically review the current literature and to analyze all studies implementing EGDT for the management of patients with severe sepsis or septic shock.

## Materials and methods

We performed this systematic review using the guidelines proposed by the Cochrane Collaboration in the Cochrane Handbook for Systematic Reviews of Interventions [16]. There was no registered protocol.

### Study selection criteria

#### Participants

This review focused on patients with severe sepsis or septic shock who received EGDT or a sepsis bundle including EGDT.

#### Interventions

For the purpose of the review, we use the term “EGDT” to describe standard EGDT, modified EGDT, or a sepsis bundle based on standard EGDT, with details presented in Table 1. Standard EGDT was described as a 6-h protocol resuscitation conforming to specific therapeutic targets of central venous pressure (CVP) between 8 and 12 mm Hg, mean arterial pressure (MAP) between 65 and 90 mm Hg, urine output 0.5 ml/kg/h or more, and continuous monitoring to keep central venous oxygen saturation (ScvO_2_) at 70% or above [4]. We defined modified EGDT as a similar or simplified 6-h protocol based on standard EGDT [4]. The intervention of the control group was usual care or other strategies described in original studies.

**Table 1:** **EGDT protocol and outcome of selected trials**

**Study**	**EGDT group**	**Control group**	**Survival benefit**
Standard EGDT versus usual care			
ARISE 2014 [13]	ScvO_2_ ≥ 70%	Usual care	No: 28d/90d/ICU/in-hospital mortality
	CVP ≥ 8-12 mm Hg		
	MAP ≥ 65 mm Hg		
	UO ≥ 0.5 ml/kg/h		
Jing 2010 [8]	ScvO_2_ ≥ 70%	CVP ≥ 8-12 mm Hg	Yes: 28d/ICU mortality
	CVP ≥ 8-12 mm Hg	SBP > 100 mm Hg	
	SBP >100 mm Hg	MAP ≥ 65 mm Hg	
	MAP ≥ 65 mm Hg	UO ≥ 0.5 ml/kg/h	
	UO ≥ 0.5 ml/kg/h		
ProCESS 2014 [14]	ScvO_2_ ≥ 70%	Usual care	No: 60d/in-hospital mortality
	CVP ≥ 8-12 mm Hg		
	MAP ≥ 65 mm Hg		
	UO ≥ 0.5 ml/kg/h		
Rivers 2001 [4]	ScvO_2_ ≥ 70%	CVP ≥ 8-12 mm Hg	Yes: 28d/60d/in-hospital mortality
	CVP ≥ 8-12 mm Hg	MAP ≥ 65 mm Hg	
	MAP ≥ 65 mm Hg	UO ≥ 0.5 ml/kg/h	
	UO ≥ 0.5 ml/kg/h		
Wang 2006 [25]	ScvO_2_ ≥ 70%	Usual care	No: 7d/14d mortality
	CVP ≥ 8-12 mm Hg		
	MAP ≥ 65 mm Hg		
	UO ≥ 0.5 ml/kg/h		
Modified EGDT versus usual care			
Andrews 2014 [21]	JVP > 3 cm;	Usual care	No: 28d/in-hospital mortality
	MAP > 65 mm Hg;		
	Hb > 7 g/dl		
Lin 2006 [23]	CVP ≥ 8-12 mm Hg;	Usual care	Yes: ICU/in-hospital mortality
	MAP ≥ 65 mm Hg;		
	UO ≥ 0.5 ml/kg/h		
ProCESS 2014 [14]	SBP ≥ 100 mm Hg	Usual care	No: 60d/in-hospital mortality
	Hb > 7.5 g/dl		
Standard EGDT versus lactate clearance			
Jones 2010 [22]	ScvO_2_ ≥ 70%	Lactate clearance ≥ 10%	No: in-hospital mortality
	CVP ≥ 8-12 mm Hg	CVP ≥ 8-12 mm Hg	
	MAP ≥ 65 mm Hg	MAP ≥ 65 mm Hg	
	UO ≥ 0.5 ml/kg/h	UO ≥ 0.5 ml/kg/h	
Wang 2014 [20]	ScvO_2_ ≥ 70%	Lactate < 2 mmol/L	No: 7d/28d mortality
	CVP ≥ 8-12 mm Hg	CVP ≥ 8-12 mm Hg	
	MAP ≥ 65 mm Hg	MAP ≥ 65 mm Hg	
	UO ≥ 0.5 ml/kg/h	UO ≥ 0.5 ml/kg/h	
Yu 2013 [24]	ScvO_2_ ≥ 70%	Lactate clearance ≥ 10%	No: 28d mortality
	CVP ≥ 8-12 mm Hg	CVP ≥ 8-12 mm Hg	
	MAP ≥ 65 mm Hg	MAP ≥ 65 mm Hg	
	UO ≥ 0.5 ml/kg/h	UO ≥ 0.5 ml/kg/h	

*Abbreviations:* *EGDT* early goal-directed therapy, *SBP* systolic blood pressure, *JVP* jugular venous pressure, *MAP* mean artery pressure, *Hb* hemoglobin, *UO* urine output.

#### Types of outcome measures

The primary outcomes were mortality among patients with severe sepsis or septic shock. Length of ICU and in-hospital stay, mechanical ventilation support, vasopressor and inotropic agents support, fluid administration and red cell transfusion rate in the first 6 h were also analyzed.

#### Types of studies

We included all RCTs comparing EGDT with usual care or other strategies for patients with severe sepsis or septic shock. We excluded non-randomized studies, studies published in abstracts, reviews, commentaries, and editorials.

### Search methods for identification of studies

#### Study selection

We used the Cochrane risk of bias tool [17] to undertake, and the PRISMA (Preferred Reporting Items for Systematic Reviews and Meta-Analyses) statement methodology [18] to report, a systematic review and meta-analysis of RCTs. Two independent reviewers (LZ and GZ) conducted a search in PubMed, Embase, the Cochrane Library, Google Scholar, a Chinese database (SinoMed), and major critical care medicine journals. Trials were considered without language or date restriction. We performed the last updated search on 5 October 2014. The following text words and corresponding heading terms were used as search terms: “sepsis or septic shock” and “EGDT or early goal directed or goal directed or goal oriented or goal target or sepsis bundle or hemodynamic optimization or protocol or program or procedure”. The exact search strategy was provided in Appendix 1. Related articles and reference lists were manually searched to avoid omissions. After title screening, we evaluated abstracts for relevance and identified them as included, excluded, or requiring further assessment. At this stage, if a paper required further assessment, we contacted the study lead investigator by e-mail and/or telephone with a request for further information.

#### Data extraction

The inclusion criteria were as follows: (a) sepsis patients with hypotension (systolic blood pressure of less than 90 mm Hg or a mean arterial pressure of less than 65 mm Hg) or hypoperfusion (blood lactate level of 4.0 mmol per liter or more); and (b) studies comparing EGDT with usual care or other intervention, and (c) sufficient data available to calculate a relative risk (RR) or mean difference (MD) with 95% confidence interval (95% CI). The following exclusion criteria were used: (a) EGDT was performed in all patients or studies of compliance with EGDT; and (b) EGDT not based on published protocol [4]; and (c) pediatric patients; and (d) nonhuman studies. For studies with the same or overlapping data by the same authors, the most suitable studies with the largest number of cases or latest publication dates were selected.

Two investigators (LZ and GZ) assessed each trial independently and recorded eligibility, quality, and outcomes. Disagreements regarding eligibility arose with 7% of the articles (κ = 0.87), which were resolved by a third party through consensus. A third investigator (FP) provided arbitration in case of disagreement. We extracted the following study features: first author, publication year, country, number of participants, protocol of EGDT, mortality, length of ICU and in-hospital stay, ventilation rate, vasopressor support, inotropic agents support, and parameters and laboratory results. Endpoints reported in three or more articles were extracted.

#### Quantitative data synthesis

Independently and in duplicate, reviewers assessed risk of bias using the Cochrane collaboration tool [17]. For each included study, a description, a comment, and a judgment as “high”, “unclear”, or “low” risk of bias were provided for each of the following domains: adequate random sequence generation; allocation sequence concealment; blinding for objective outcomes; incomplete outcome data; free of selective outcome reporting; free of other bias. Studies with high risk of bias for any one or more key domains were considered as at high risk of bias. Studies with low risk of bias for all key domains were considered as at low risk of bias. Otherwise, they were considered as unclear risk of bias.

Before the analysis, data were standardized into equivalent units. For dichotomous variables such as mortality, the rates in the experimental (EGDT) and control groups were expressed as RR and 95% CI. For continuous variables such as length of ICU stay, MD and 95% CI were calculated for each study. Heterogeneity was evaluated using the Mantel-Haenszel chi-square test and the *I*^2^ statistic to assess the degree of interstudy variation. When statistically significant heterogeneity was detected with a *P* value less than 0.10, a pooled analysis of each study was performed in the random-effects model. Also since the chi-square Cochran Q test for heterogeneity assessment is underpowered, a *P* value of 0.10 should be considered as a threshold.

Publication bias was analyzed once sufficient RCTs were identified, by visual inspection of asymmetry in funnel plots as well as the Egger’s test [19]. Sensitivity analysis was conducted by sequentially deleting a single study each time in an attempt to identify the potential influence of an individual study. Data analysis was performed using Review Manager 5.2 (RevMan, The Cochrane Collaboration, Oxford, United Kingdom) and STATA 12.0 (StataCorp, College Station, TX, USA).

## Results

### Eligible studies

**Figure 1:**
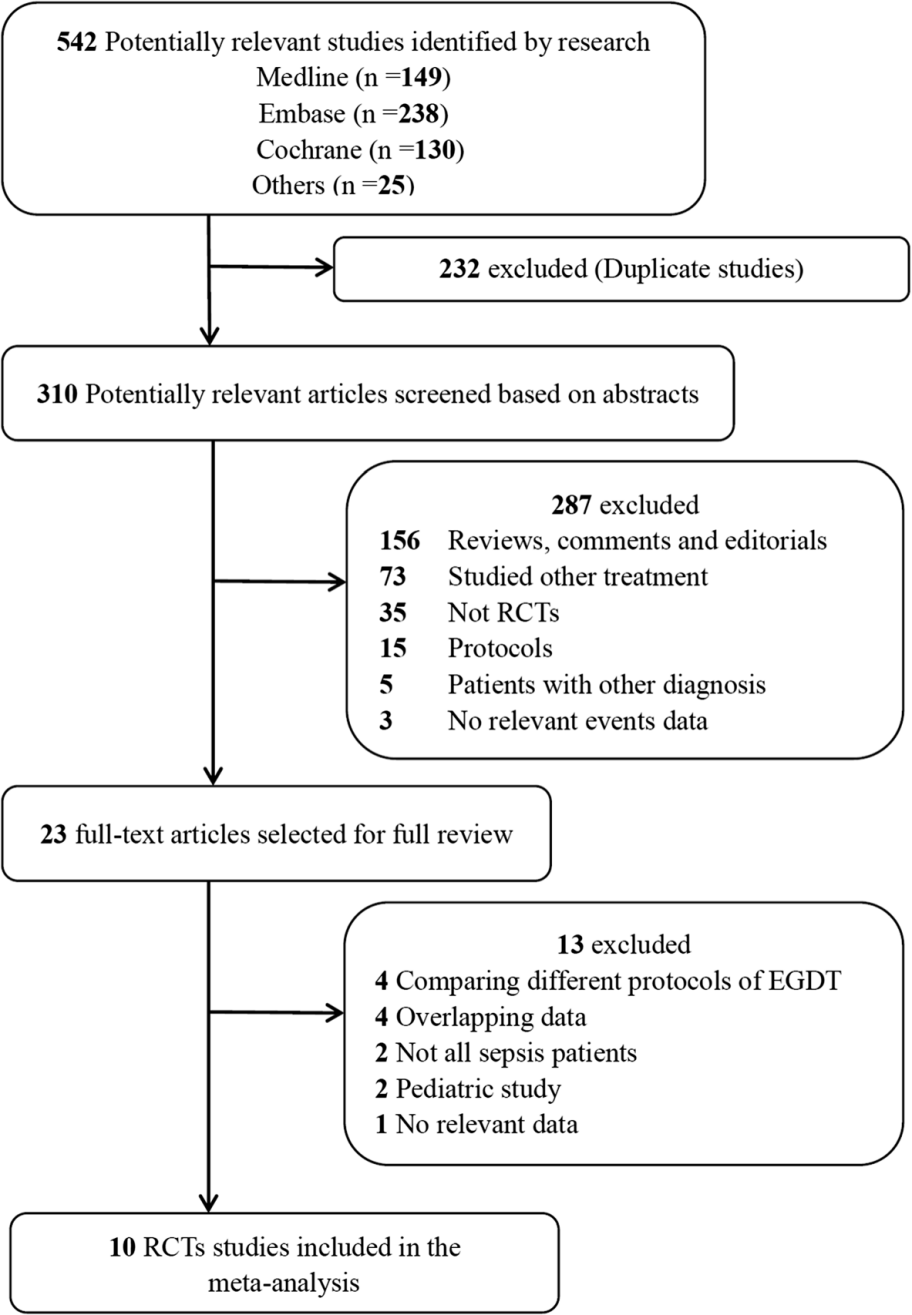
**Flow chart of selection of studies.**

The study selection process is presented in Figure 1. The literature search yielded 542 potentially relevant records. By screening the titles, we removed 232 duplicate studies. After evaluating the abstract of each, 287 studies were excluded as they did not meet the inclusion criteria. Subsequently, we carefully read the full text of each of the remaining 23 trials and excluded 13 trials: we compared different protocols of EGDT (n = 4); overlapping data (n = 4); not all sepsis patients (n = 2); pediatric study (n = 2), and no relevant data (n = 1). Finally, 10 RCTs [4,8,13,14,20-25] comparing EGDT with other interventions for severe sepsis or septic shock were included. Among the included RCTs, 5 studies compared standard EGDT with usual care [4,8,13,14,25], 3 compared modified EGDT (not monitoring ScvO_2_) with usual care [14,21,23], and 3 compared standard EGDT with early lactate clearance [20,22,24].

The eligible studies were conducted from 2001 to 2014 with a total number of 2,280 patients in EGDT and 1,877 in other interventions. There were 5 studies from Asia, 3 from North America, 1 from Oceania, and 1 from Africa. A variety of outcomes were recorded in these studies, including mortality (10 studies) [4,8,13,14,20-25], 28-d mortality (6 studies) [4,8,13,20,21,24], in-hospital mortality (6 studies) [4,13,14,21-23], length of ICU stay (6 studies) [8,14,20,22-24], length of in-hospital stay (4 studies) [14,22-24], ventilation rate (5 studies) [4,13,14,22,25], ventilation days (4 studies) [4,8,20,23], vasopressor support (6 studies) [4,13,14,21-23], inotropic agents support (5 studies) [4,13,14,22,23], fluid administration in the first 6 h (7 studies) [4,13,14,21,22,24,25], and red cell transfusion rate in the first 6 h [4,13,14,22,24]. The characteristics of the RCT studies fulfilling the inclusion criteria are listed in Table 2.

**Table 2:** **Baseline characteristics of selected trials of EGDT in severe sepsis or septic shock**

**Study**	**Country**	**N**	**Male (%)**	**Age (y)**	**Center**	**Illness severity scores**	**Overall risk of bias**
Andrews 2014 [21]	Zambia	109	53.2	35.2, 34.8	S	APACHE II: 17.8, 17.9	Low
ARISE 2014 [13]	Australia/New Zealand	1,588	59.8	62.7, 63.1	M	APACHE II: 15.4, 15.8	Low
Jing 2010 [8]	China	317	69.3	68.9, 67.7	M	APACHE II: 23.5, 21.8	Low
Jones 2010 [22]	USA	300	54.3	59.8, 61.6	M	SAPS II: 44.8, 44.1	Low
Lin 2006 [23]	Taiwan	224	58.0	67.2, 68.7	S	APACHE III: 66.5, 64.9	Low
ProCESS 2014 [14]	USA	1,341	55.4	60, 62	M	APACHE II: 20.8, 20.7	Low
Rivers 2001 [4]	USA	263	50.6	67.1, 64.4	S	APACHE II: 20.4, 21.4	Low
Wang 2006 [25]	China	33	NA	33, 36	S	APACHE II: 28, 27	Unclear
Wang 2014 [20]	China	57	70.2	52, 56	S	APACHE II: 19.7, 20.9	Unclear
Yu 2013 [24]	China	50	74.0	61, 59	S	APACHE II: 18.2, 17.9	Unclear

*Abbreviations:* *N* number of patients, *y* *year* S, single center, *M* multicenter, *APACHE* Acute Physiology and Chronic Health Evaluation, *SAPS* Simplified Acute Physiology Score.

### Assessment of methodological quality

**Figure 2:**
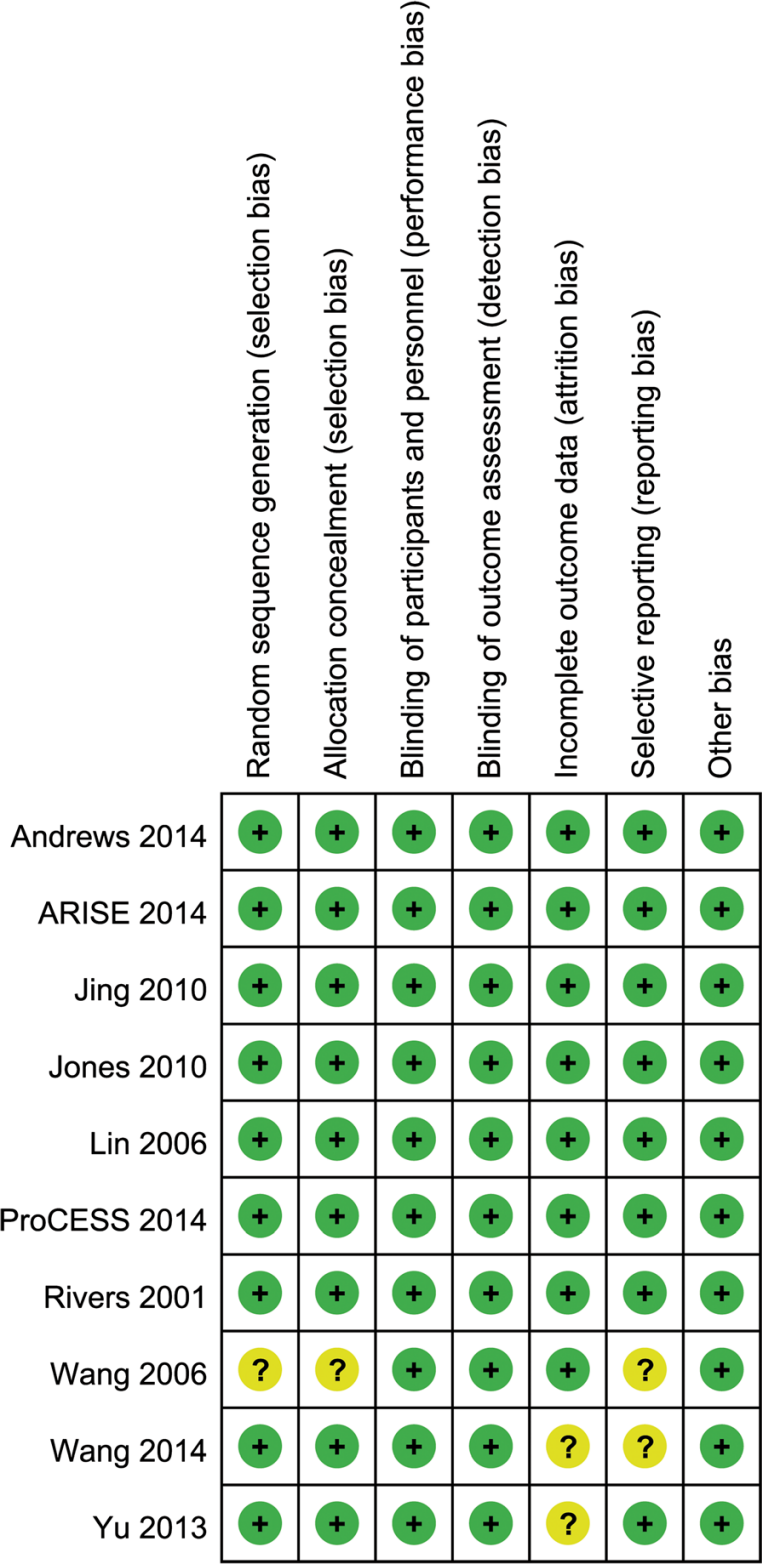
**Risk of bias summary.**

The details of risk of bias are summarized in Figure 2. Seven studies were judged to be at low risk of bias, and the other three were judged to be at unclear risk of bias. Nine trials generated adequate randomized sequences and reported appropriate allocation concealment [4,8,13,14,21-23]. Among all RCTs, none of them were double-blinded. However, blinding of patients and clinicians was extremely difficult in these studies to evaluate a complex intervention such as EGDT protocol, and the authors judged that the primary outcome (mortality) is not likely to be influenced by lack of blinding.

### Mortality

**Figure 3:**
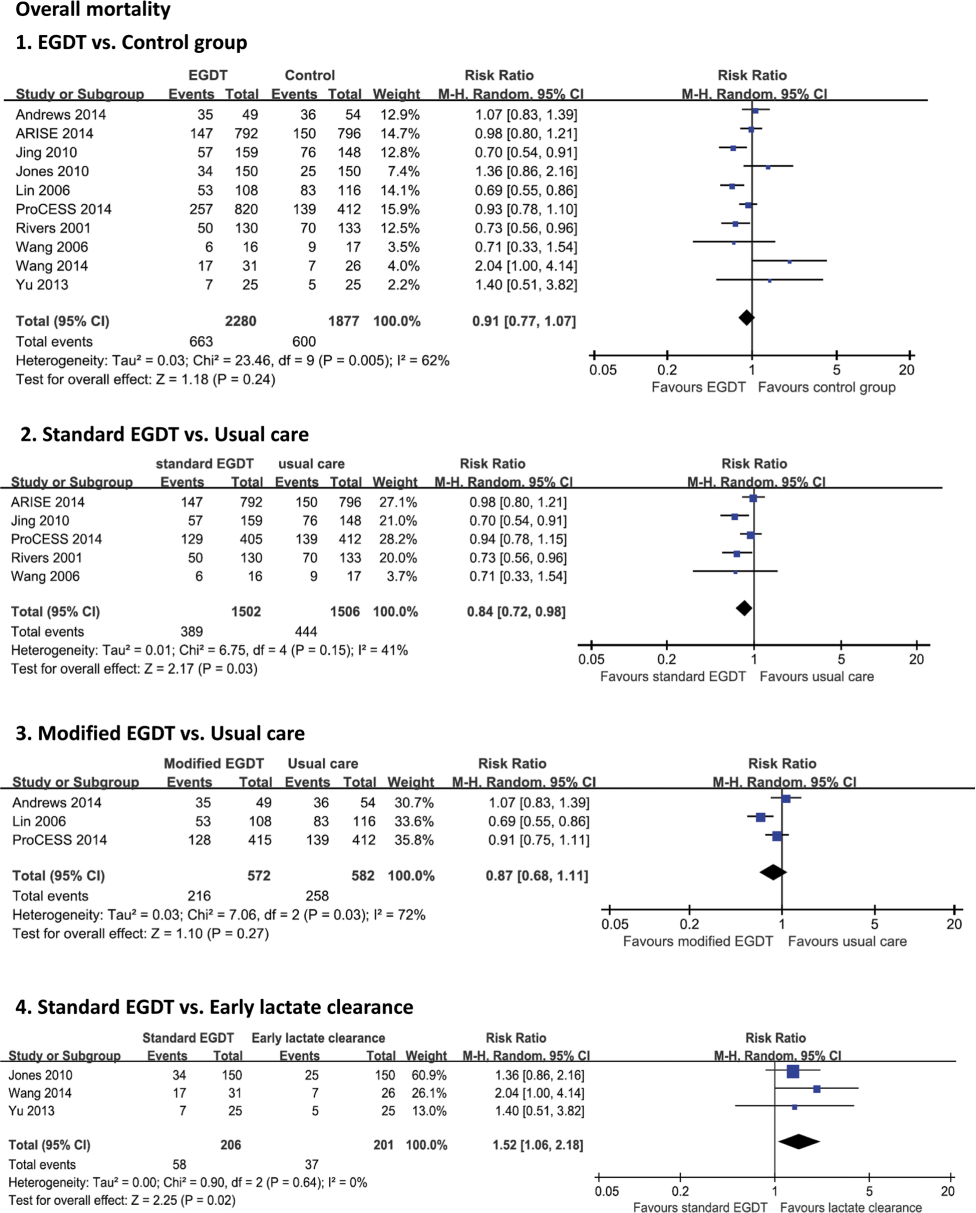
**Forest plot for overall mortality.** The analysis was stratified by study design. Risk ratio (RR) < 1.0 favors EGDT. Abbreviations: CI, confidence interval; M-H, Mantel-Haenszel.

**Figure 4:**
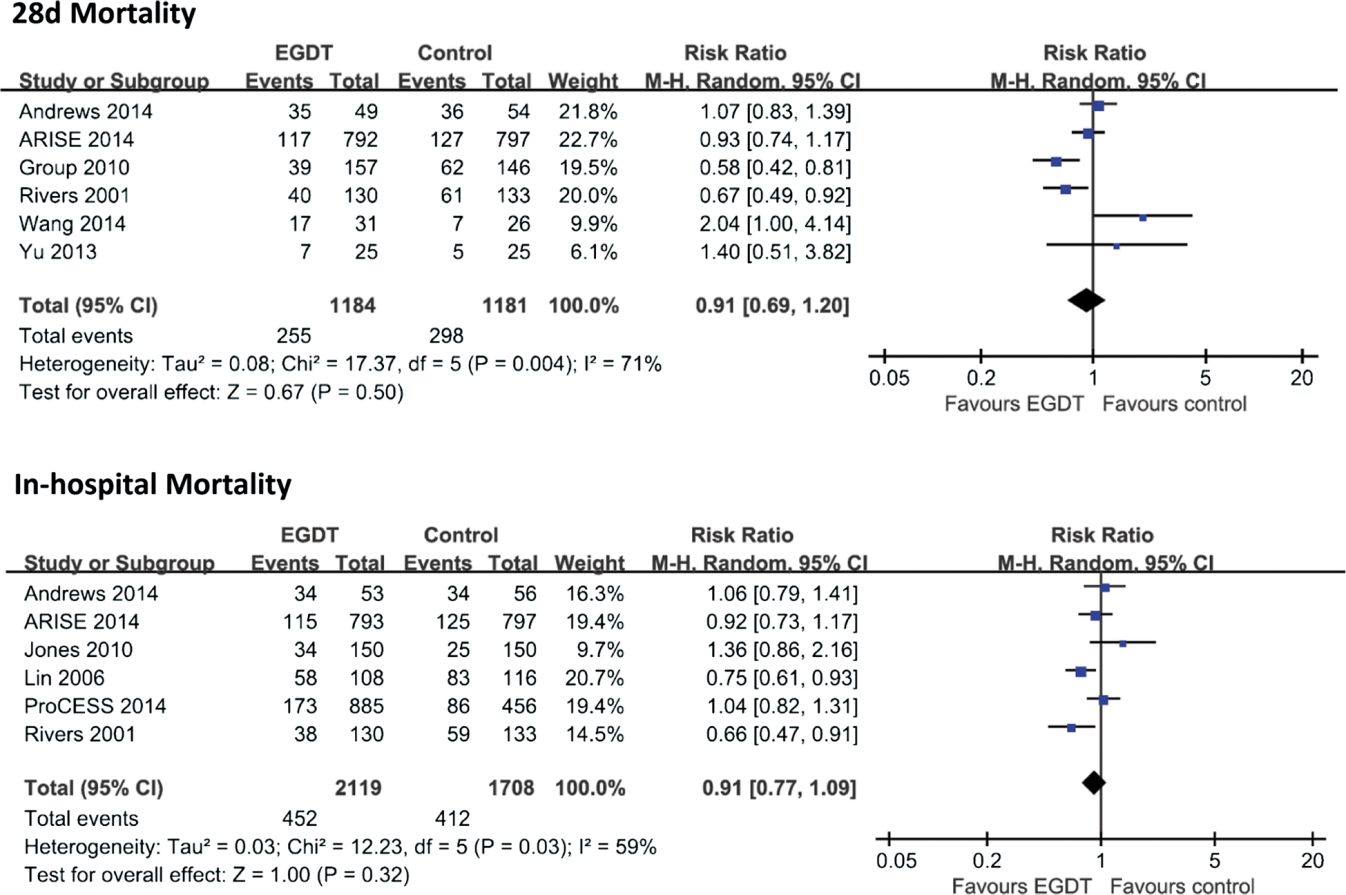
**Forest plot for 28-d mortality and in-hospital mortality.** The analysis was stratified by study design. Risk ratio (RR) < 1.0 favors EGDT. Abbreviations: CI, confidence interval; M-H, Mantel-Haenszel.

A total of 10 RCTs including 4,157 patients reported data on mortality. Overall mortality was 30.4%. Of the EGDT group, 29.1% of patients died compared with 32.0% in the control group. As shown in Figure 3, pooled analyses of all studies showed that there was no significant difference in mortality between the EGDT group and the control group (RR 0.91, 95% CI: 0.77 to 1.07, *P* = 0.24), with substantial heterogeneity (*χ*2 = 23.46, *I*^2^ = 62%). There was also no significant difference in 28-d mortality (RR 0.91, 95% CI: 0.69 to 1.20, *P* = 0.50) or in-hospital mortality (RR 0.91, 95% CI: 0.77 to 1.09, *P* = 0.32) (Figure 4). In the subgroup analysis, standard EGDT (5 studies including 3,004 patients), but not modified EGDT, was associated with a lower mortality rate in comparison to the usual care group (RR 0.91, 95% CI: 0.72 to 0.98, *P* = 0.03) (*I*^2^ = 42%). However, EGDT (3 studies including 407 patients) was associated with a higher mortality rate in comparison to the early lactate clearance group (RR 1.52, 95% CI: 1.06 to 2.18, *P* = 0.02) (*I*^2^ = 0%).

### Length of ICU and in-hospital stay

A total of 6 RCTs including 1,829 patients provided information on length of ICU stay. As shown in Table 3, a *Z*-test in a random-effects model showed no significant difference in length of ICU stay (d) with EGDT in comparison to the control group (MD -0.20 d, 95% CI: -1.31 to 0.92; *P* = 0.73). There was considerable evidence of heterogeneity (*χ*2 = 20.23, *I*^2^ = 75%).

**Table 3:** **Pooled analysis of secondary outcomes**

**Outcome**	**Comparison**	**Number of studies**	**MD or RR (95% CI)**	***P***	***I*** ^**2**^
Length of ICU stay (d)	EGDT versus control group	6	-0.20 (-1.31 to 0.92)	0.73	75%
Length of in-hospital stay (d)	EGDT versus control group	4	0.42 (-1.02 to 1.86)	0.57	12%
Mechanical ventilation rate	EGDT versus control group	5	0.96 (0.85 to 1.09)	0.53	43%
Mechanical ventilation days (d)	EGDT versus control group	4	-0.91 (-2.34 to 0.52)	0.21	95%
Vasopressor support rate	EGDT versus control group	6	1.03 (0.93 to 1.15)	0.58	69%
Inotropic agents support	EGDT versus control group	5	2.23 (1.06 to 4.67)	0.03	84%
	EGDT versus usual care group	4	2.37 (1.02 to 5.51)	0.05	
	EGDT versus early lactate clearance	1	1.60 (0.54 to 4.78)	0.40	88%-
Fluid administration in first 6 h (L)	EGDT versus control group	7	0.88 (-0.17 to 1.93)	0.10	99%
	EGDT versus usual care group	5	1.24 (0 to 2.48)	0.05	99%
	EGDT versus early lactate clearance	2	0.02 (-0.46 to 0.49)	0.27	17%
Red cell transfusion rate in first 6 h	EGDT versus control group	5	1.76 (1.11 to 2.78)	0.04	76%
	EGDT versus usual care group	3	2.26 (1.54 to 3.31)	<0.01	71%
	EGDT versus early lactate clearance	2	0.72 (0.27 to 1.94)	0.52	37%

*Abbreviations:* *EGDT* early goal-directed therapy, *MD* mean difference, *RR* relative risk.

A total of 4 RCTs including 1,469 patients described data on length of in-hospital stay (d) with no evidence of heterogeneity (*χ*2 = 3.41, *I*^2^ = 12%). There was no significant difference in length of in-hospital stay between EGDT and the control group (MD 0.42 d, 95% CI: -1.02 to 1.86; *P* = 0.33) (Table 3).

### Mechanical ventilation support

In Table 3, there were 5 studies including 3,082 patients that provided information on mechanical ventilation rate. No significant difference in mechanical ventilation rate was found between EGDT and the control group (RR 0.96, 95% CI: 0.85 to 1.09; *P* = 0.53), and there was moderate evidence of heterogeneity (*χ*2 = 7.07, *I*^2^ = 43%).

A total of 4 studies including 847 patients reported data on mechanical ventilation days (d) with considerable heterogeneity (*χ*2 = 56.77, *I*^2^ = 95%). There was no significant difference in ventilation days with EGDT in comparison to the control group (MD -0.91 d, 95% CI: -2.34 to 0.52; *P* = 0.21).

### Vasopressor and inotropic agents support

There were 6 RCTs including 3,828 patients that described data on vasopressor support rate, and there was substantial heterogeneity (*χ*2 = 16.19, *I*^2^ = 69%, *P* < 0.01). There was no significant difference in vasopressor support rate between EGDT and the control group (RR 1.03, 95% CI: 0.93 to 1.15; *P* = 0.58) (Table 3).

Overall, 5 RCTs including 3,273 patients provided information on inotropic agents support rate. EGDT was associated with higher inotropic agents support rate in comparison to the control group (RR 2.23, 95% CI: 1.06 to 4.67; *P* = 0.03). There was considerable evidence for heterogeneity (*χ*2 = 25.30, *I*^2^ = 84%). In a subgroup analysis, patients in EGDT received more inotropic agents in comparison to the usual care group (RR 2.37, 95% CI: 1.02 to 5.51; *P* =0.05), whereas the results between the EGDT group and the early lactate clearance group were not significant (*P* = 0.40).

### Fluid administration and red cell transfusion rate in first 6 h

As shown in Table 3, a total of 7 studies including 3,204 patients provided information on fluid administration (L) in the first 6 h with considerable heterogeneity (*χ*2 = 788.12, *I*^2^ = 99%). There was no significant difference between EGDT and the control group (MD 0.88 L, 95% CI: -1.07 to 1.93; *P* = 0.10). In a subgroup analysis, EGDT was associated with more fluid administration in the first 6 h compared with the usual care group (MD 1.24 L, 95% CI: 0 to 2.48; *P* = 0.05), whereas results between the EGDT group and the early lactate clearance group were not significant (*P* = 0.27).

A total of 5 studies including 3,097 patients reported data on red cell transfusion rate in the first 6 h with considerable heterogeneity (*χ*2 = 16.49, *I*^2^ = 76%). EGDT was associated with a higher red cell transfusion rate in comparison to the control group (RR 1.76, 95% CI: 1.11 to 2.78; *P* = 0.04). There was also a significant difference between EGDT and the usual care group (RR 2.26, 95% CI: 1.54 to 3.31; *P* < 0.01). No significant difference was found between the EGDT group and the early lactate clearance group (*P* = 0.52).

### Publication Bias

**Figure 5:**
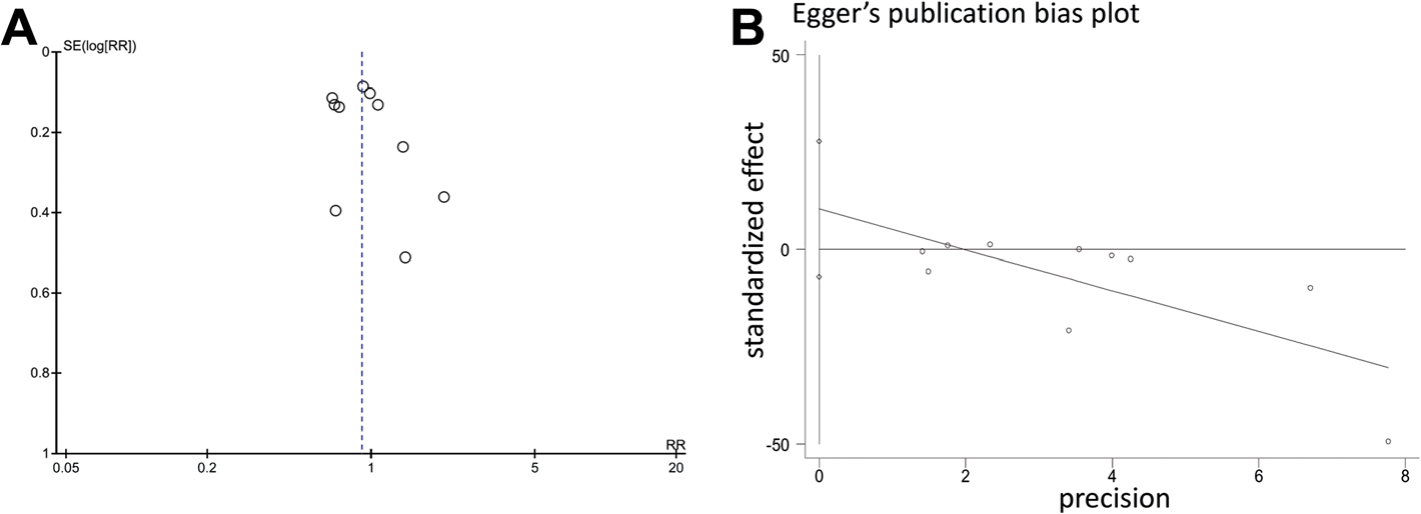
**Assessment of publication bias. (A)** funnel plot. **(B)** Egger’s test. Abbreviations: SE, standard error, RR, risk ratio.

No evidence of publication bias was detected for RR of mortality by either funnel plots or Egger’s test (*t* = 1.37; *P* = 0.209) (Figure 5).

### Sensitivity analysis

In order to assess the stability of the results of the current meta-analysis, we performed a sensitivity analysis for each outcome by removing a study. Statistically similar results were obtained after omitting each of the studies (Table 4), indicating a moderate degree of stability in the findings of this systematic review.

**Table 4:** **Sensitivity analysis for mortality by omitting each study in random-effects model**

**Study omitted**	**RR (95% CI)**	***P*** **value**
Andrews [21]	0.89 (0.75 to 1.06)	0.19
ARISE [13]	0.90 (0.75 to 1.08)	0.27
Jing [8]	0.94 (0.80 to 1.12)	0.50
Jones [22]	0.88 (0.75 to 1.03)	0.11
Lin [23]	0.95 (0.80 to 1.11)	0.50
ProCESS [14]	0.91 (0.75 to 1.11)	0.37
Rivers [4]	0.94 (0.79 to 1.12)	0.47
Wang [20]	0.92 (0.78 to 1.08)	0.31
Wang [20]	0.87 (0.76 to 1.01)	0.07
Yu [24]	0.90 (0.76 to 1.06)	0.20

## Discussion

### Key findings

We performed a systematic review of the literature and identified 10 RCTs reporting data on EGDT versus control group among more than 3,700 patients with severe or septic shock. We found that patients receiving EGDT had a similar risk of mortality compared with those in the control group. In a subgroup analysis, a difference in favor of standard EGDT was seen in comparison to the usual care group. However, EGDT was associated with a higher rate of mortality compared with the early lactate clearance group. In a first 6 h-protocol of EGDT, compared with usual care, patients in EGDT received more inotropic agents support, fluid administration, and blood transfusion. No significant differences were found in length of ICU stay or in-hospital stay, ventilation rate, ventilation days, and vasopressor support between EGDT and the control group.

### Comparison with previous studies

As shown in Table 5, there were a few meta-analysis studies to evaluate the effect of EGDT or a 6-h sepsis bundle including EGDT on patients with severe sepsis or septic shock [12,26-28]. All four previous meta-analyses showed that EGDT was associated with a survival benefit. However, there were some problems with these meta-analyses. Among them, the latest meta-analysis [28], included 13 RCTs, but only 7 studies in the EGDT subgroup; second, some protocols which differed from the original one and that recommended by the surviving sepsis campaign guidelines were included [29-32]; third, two inappropriate RCTs were included: one was not an RCT but a before-and-after study, and the other included non-sepsis patients with no information about mortality of sepsis subgroup; fourth, studies about EGDT comparing it with early lactate clearance were not included [20,22,24]; fifth, the latest ARISE study [13] was not included.

**Table 5:** **Comparison of our study with previous meta-analyses**

	**Our study**	**Gu** [28]	**Wira** [26]	**Chamberlain** [27]	**Barochia** [12]
Year of publication		2014	2014	2011	2010
Years of searching	1966-2014	NA-2014	1980-2011	2004-2010	1980-2008
Key finding	EGDT	GDT	EGDT	6-h sepsis bundle	6 h sepsis bundle
Studies included	10	13	25	11	8
*RCTs*	10	13	1	0	1
*Observational studies*	0	0	14	11	7
*Abstracts*	0	0	10	0	0
Survival benefit	Negative	Favors GDT	Favors EGDT	Favors EGDT	Favours EGDT

*Abbreviations:* *EGDT* early goal-directed therapy, *GDT* goal-directed therapy.

In the other three previous meta-analyses, the recent trials were not included. The positive findings largely relied on data from observational studies, so potential selection and allocation bias acted as major confounders. Second, some inappropriate studies which evaluated compliance with EGDT or 6-h sepsis bundles were included [33,34]. Third, mortality rates of severe sepsis or septic shock have dropped year by year over time [35,36]. However, all the included observational studies used a before-and-after design, and the patients in the EGDT group were treated 1 to 2 years later than those in the control group, introducing a time bias.

In contrast, the present systematic review includes data from 10 RCTs with more than 3,700 patients with severe sepsis and septic shock. Such studies might be more likely to accurately represent the efficacy of EGDT on patients with severe sepsis and septic shock. An ongoing multicenter RCT (ProMISe, ISRCTN36307479) [37] in the United Kingdom comparing EGDT with usual care for severe sepsis or septic shock will provide us more information in the future.

### Clinical implications and future studies

Among the RCTs included in the present systematic review, five recent studies (after 2013) [13,14,20,21,24] showed no survival benefit with EGDT for patients with severe sepsis or septic shock, which indicates that the efficacy of EGDT should be reevaluated. Compared with usual care, continuous monitoring of ScvO_2_, which requires invasive central venous catheterization and special equipment, is the key method of EGDT. However, its effectiveness is still uncertain [38]. In contrast, it is convenient to monitor lactate levels, and early lactate clearance may be more effective for severe sepsis or septic shock than EGDT in the present meta-analysis. A recent multicenter RCT [39] also reported that early lactate-guided therapy significantly reduced hospital mortality in critically ill patients with hyperlactatemia; however, it was not included in the meta-analysis because non-sepsis patients were enrolled in the study. Thus, future studies should focus on comparing EGDT with early lactate clearance as a therapeutic option in severe sepsis or septic shock.

### Strengths and limitations

To the best of our knowledge, this study is the first to systematically evaluate the effect of EGDT on patients with severe sepsis or septic shock based on RCTs. Our search strategy was broad and included studies in both English and Chinese. It included data from more than 3,700 patients, 10 RCTs, and 6 countries, from different regions of Asia, North America, Oceania, and Africa. Two independent investigators also rigorously assessed methodological quality.

However, our study also has several limitations. First, although 10 studies were included in this systematic review, three of the included studies were small (less than 60 patients). There was moderate evidence for heterogeneity in main outcomes such as mortality. Subgroup analysis was performed to solve this when data were available, but subgroup analysis in a meta-analytical study can only provide weak hypothesis-generating evidence. Thus, we do not believe that these results constitute a reason to change clinical practice but rather support the need for further research.

Second, because of the nature of the intervention and logistic problem, the studies were not double-blinded. Although it might not influence the primary outcome (mortality), there is still potential for bias.

Third, although we extracted data on mortality at the end of follow-up, the duration of each study varied from 14 days in one study [25], to 28 days in 3 studies [8,20,24], to in-hospital mortality in 6 studies [4,13,14,21-23]. Even so, although the end points of different follow-up periods could modify the absolute risk, they should not bias the overall RR.

Fourth, the variation in baseline among studies might also be a contributing factor to clinical and possibly statistical heterogeneity. For instance, the APACHE II score and total mortality in the ARISE study and River’s study were 15, 18.7% and 20, 45.6%, respectively. In addition, the intervention in the control group (usual care group) was not clear and might be different among studies.

Last but not least, only published studies with selective databases were included for data analysis. The unavailability of unreported outcomes possibly could result in reporting bias. Regardless of these limitations, we have minimized bias throughout the process by our methods of study identification, data selection, and statistical analysis, as well as in our control of publication bias and sensitivity. These steps should strengthen the stability and accuracy of the meta-analysis.

## Conclusions

Available RCTs do not show a significant difference in mortality between the EGDT group and the control group. In subgroup analysis, EGDT is associated with a lower mortality rate in comparison to the usual care group. However, EGDT was associated with a higher mortality rate in comparison to the early lactate clearance group. Further high-quality RCTs comparing EGDT and early lactate clearance are desirable.

## Appendix 1: Electronic search strategies

1. Medline
  1. sepsis.ti,ab,kw.
  2. septic shock.ti,ab,kw.
  3. sepsis/
  4. 1 or 2 or 3
  5. EGDT.ti,ab,kw.
  6. goal directed.ti,ab,kw.
  7. goal oriented.ti,ab,kw.
  8. bundle.ti,ab,kw.
  9. hemodynamic optimization.ti,ab,kw.
  10. Resuscitation.ti.
  11. protocol.ti.
  12. program.ti.
  13. procedure.ti.
  14. 5 or 6 or 7 or 8 or 9 or 10 or 11 or 12 or 13
  15. 4 and 13
  16. random.ti,ab,kw.
  17. randomly.ti,ab,kw.
  18. randomized.ti,ab,kw.
  19. 16 or 17 or 18
  20. 15 and 19
  21. limit 20 to humans
2. Embase
  1. sepsis.ti,ab,kw.
  2. septic shock.ti,ab,kw.
  3. sepsis/
  4. 1 or 2 or 3
  5. EGDT.ti,ab,kw.
  6. goal directed.ti,ab,kw.
  7. goal oriented.ti,ab,kw.
  8. bundle.ti,ab,kw.
  9. hemodynamic optimization.ti,ab,kw.
  10. Resuscitation.ti.
  11. protocol.ti.
  12. program.ti.
  13. procedure.ti.
  14. 5 or 6 or 7 or 8 or 9 or 10 or 11 or 12 or 13
  15. 4 and 13
  16. random.ti,ab,kw.
  17. randomly.ti,ab,kw.
  18. randomized.ti,ab,kw.
  19. 16 or 17 or 18
  20. 15 and 19
  21. limit 20 to humans
3. Cochrane Library
  1. sepsis.ti,ab,kw.
  2. septic shock.ti,ab,kw.
  3. 1 or 2
  4. EGDT.ti,ab,kw.
  5. goal directed.ti,ab,kw.
  6. goal oriented.ti,ab,kw.
  7. bundle.ti,ab,kw.
  8. hemodynamic optimization.ti,ab,kw.
  9. Resuscitation.ti.
  10. protocol.ti.
  11. program.ti.
  12. procedure.ti.
  13. 4 or 5 or 6 or 7 or 8 or 9 or 10 or 11 or 12
  14. 3 and 13
  15. random.ti,ab,kw.
  16. randomly.ti,ab,kw.
  17. randomized.ti,ab,kw.
  18. 15 or 16 or 17
  19. 14 and 18

## Abbreviations

APACHE: Acute Physiology and Chronic Health Evaluation
CVP: central venous pressure
EGDT: early goal-directed therapy
Hb: hemoglobin
JVP: jugular venous pressure
MAP: mean arterial pressure
MD: mean difference
RCT: randomized controlled trials
RR: relative risk
SAPS: Simplified Acute Physiology Score
SBP: systolic blood pressure
ScvO_2_: central venous oxygen saturation
UO: urine output

## References

[1] Angus DC, Linde-Zwirble WT, Lidicker J, Clermont G, Carcillo J, Pinsky MR (2001). Epidemiology of severe sepsis in the United States: analysis of incidence, outcome, and associated costs of care. Crit Care Med..

[2] Dombrovskiy VY, Martin AA, Sunderram J, Paz HL (2007). Rapid increase in hospitalization and mortality rates for severe sepsis in the United States: a trend analysis from 1993 to 2003. Crit Care Med..

[3] Martin GS, Mannino DM, Eaton S, Moss M (2003). The epidemiology of sepsis in the United States from 1979 through 2000. N Engl J Med..

[4] Rivers E, Nguyen B, Havstad S, Ressler J, Muzzin A, Knoblich B (2001). Early goal-directed therapy in the treatment of severe sepsis and septic shock. N Engl J Med..

[5] Dellinger RP, Carlet JM, Masur H, Gerlach H, Calandra T, Cohen J (2004). Surviving Sepsis Campaign guidelines for management of severe sepsis and septic shock. Crit Care Med..

[6] Dellinger RP, Levy MM, Carlet JM, Bion J, Parker MM, Jaeschke R (2008). Surviving Sepsis Campaign: international guidelines for management of severe sepsis and septic shock: 2008. Crit Care Med..

[7] Dellinger RP, Levy MM, Rhodes A, Annane D, Gerlach H, Opal SM (2013). Surviving Sepsis Campaign: international guidelines for management of severe sepsis and septic shock: 2012. Crit Care Med..

[8] Jing Y (2010). The effect of early goal-directed therapy on treatment of critical patients with severe sepsis/septic shock: a multi-center, prospective, randomized, controlled study. Zhongguo Wei Zhong Bing Ji Jiu Yi Xue..

[9] Micek ST, Roubinian N, Heuring T, Bode M, Williams J, Harrison C (2006). Before-after study of a standardized hospital order set for the management of septic shock. Crit Care Med..

[10] Shapiro NI, Howell MD, Talmor D, Lahey D, Ngo L, Buras J (2006). Implementation and outcomes of the Multiple Urgent Sepsis Therapies (MUST) protocol. Crit Care Med..

[11] Puskarich MA, Marchick MR, Kline JA, Steuerwald MT, Jones AE (2009). One year mortality of patients treated with an emergency department based early goal directed therapy protocol for severe sepsis and septic shock: a before and after study. Crit Care..

[12] Barochia AV, Cui X, Vitberg D, Suffredini AF, O’Grady NP, Banks SM (2010). Bundled care for septic shock: an analysis of clinical trials. Crit Care Med..

[13] Arise Investigators (2014). Goal-directed resuscitation for patients with early septic shock. N Engl J Med.

[14] Yealy DM, Kellum JA, Huang DT, Barnato AE, Weissfeld LA, Pike F (2014). A randomized trial of protocol-based care for early septic shock. N Engl J Med.

[15] Kelm DJ, Perrin JT, Cartin-Ceba R, Gajic O, Schenck L, Kennedy CC (2014). Fluid overload in patients with severe sepsis and septic shock treated with early-goal directed therapy is associated with increased acute need for fluid-related medical interventions and hospital death. Shock.

[16] Higgins J, Green S. Cochrane handbook for systematic reviews of interventions. Version 5.1.0 [updated March 2011]. The Cochrane Collaboration. 2011. (http://www.cochrane-handbook.org).

[17] Higgins J, Altman DG, Gøtzsche PC, Jüni P, Moher D, Oxman AD (2011). The Cochrane Collaboration’s tool for assessing risk of bias in randomised trials. BMJ..

[18] Moher D, Liberati A, Tetzlaff J, Altman DG, Group P (2009). Preferred reporting items for systematic reviews and meta-analyses: the PRISMA statement. BMJ..

[19] Egger M, Davey Smith G, Schneider M, Minder C (1997). Bias in meta-analysis detected by a simple, graphical test. BMJ..

[20] Wang T, Xia Y, Hao D, Sun J, Li Z, Han S (2014). The significance of lactic acid in early diagnosis and goal-directed therapy of septic shock patients. Zhongguo Wei Zhong Bing Ji Jiu Yi Xue..

[21] Andrews B, Muchemwa L, Kelly P, Lakhi S, Heimburger DC, Bernard GR (2014). Simplified severe sepsis protocol: a randomized controlled trial of modified early goal-directed therapy in Zambia. Crit Care Med.

[22] Jones AE, Shapiro NI, Trzeciak S, Arnold RC, Claremont HA, Kline JA (2010). Lactate clearance vs central venous oxygen saturation as goals of early sepsis therapy: a randomized clinical trial. JAMA..

[23] Lin SM, Huang CD, Lin HC, Liu CY, Wang CH, Kuo HP (2006). A modified goal-directed protocol improves clinical outcomes in intensive care unit patients with septic shock: a randomized controlled trial. Shock..

[24] Yu B, Tian HY, Hu ZJ, Zhao C, Liu LX, Zhang Y (2013). Comparison of the effect of fluid resuscitation as guided either by lactate clearance rate or by central venous oxygen saturation in patients with sepsis. Zhongguo Wei Zhong Bing Ji Jiu Yi Xue..

[25] Wang XZ, Lu CJ, Gao FQ, Li XH, Yan WF, Ning FY (2006). Efficacy of goal-directed therapy in the treatment of septic shock. Zhongguo Wei Zhong Bing Ji Jiu Yi Xue..

[26] Wira CR, Dodge K, Sather J, Dziura J (2014). Meta-analysis of protocolized goal-directed hemodynamic optimization for the management of severe sepsis and septic shock in the emergency department. West J Emerg Med..

[27] Chamberlain DJ, Willis EM, Bersten AB (2011). The severe sepsis bundles as processes of care: a meta-analysis. Aust Crit Care..

[28] Gu WJ, Wang F, Bakker J, Tang L, Liu JC (2014). The effect of goal-directed therapy on mortality in patients with sepsis - earlier is better: a meta-analysis of randomized controlled trials. Crit Care..

[29] Tuchschmidt J, Fried J, Astiz M, Rackow E (1992). Elevation of cardiac output and oxygen delivery improves outcome in septic shock. Chest..

[30] Yu M, Levy MM, Smith P, Takiguchi SA, Miyasaki A, Myers SA (1993). Effect of maximizing oxygen delivery on morbidity and mortality rates in critically ill patients: a prospective, randomized, controlled study. Crit Care Med..

[31] Hayes MA, Timmins AC, Yau EH, Palazzo M, Hinds CJ, Watson D (1994). Elevation of systemic oxygen delivery in the treatment of critically ill patients. N Engl J Med..

[32] Alia I, Esteban A, Gordo F, Lorente JA, Diaz C, Rodriguez JA (1999). A randomized and controlled trial of the effect of treatment aimed at maximizing oxygen delivery in patients with severe sepsis or septic shock. Chest..

[33] Gao F, Melody T, Daniels DF, Giles S, Fox S (2005). The impact of compliance with 6-hour and 24-hour sepsis bundles on hospital mortality in patients with severe sepsis: a prospective observational study. Crit Care..

[34] Nguyen HB, Corbett SW, Steele R, Banta J, Clark RT, Hayes SR (2007). Implementation of a bundle of quality indicators for the early management of severe sepsis and septic shock is associated with decreased mortality. Crit Care Med..

[35] Stevenson EK, Rubenstein AR, Radin GT, Wiener RS, Walkey AJ (2014). Two decades of mortality trends among patients with severe sepsis: a comparative meta-analysis*. Crit Care Med..

[36] Kaukonen KM, Bailey M, Suzuki S, Pilcher D, Bellomo R (2014). Mortality related to severe sepsis and septic shock among critically ill patients in Australia and New Zealand, 2000–2012. JAMA..

[37] Power GS, Harrison DA, Mouncey PR, Osborn TM, Harvey SE, Rowan KM (2013). The Protocolised Management in Sepsis (ProMISe) trial statistical analysis plan. Crit Care Resusc..

[38] Chung KP, Chang HT, Huang YT, Liao CH, Ho CC, Jerng JS (2012). Central venous oxygen saturation under non-protocolized resuscitation is not related to survival in severe sepsis or septic shock. Shock..

[39] Jansen TC, van Bommel J, Schoonderbeek FJ, Sleeswijk Visser SJ, van der Klooster JM, Lima AP (2010). Early lactate-guided therapy in intensive care unit patients: a multicenter, open-label, randomized controlled trial. Am J Respir Crit Care Med..

